# High rates of unplanned interruptions from HIV care early after antiretroviral therapy initiation in Nigeria

**DOI:** 10.1186/s12879-015-1137-z

**Published:** 2015-09-30

**Authors:** Aimalohi A. Ahonkhai, Bolanle Banigbe, Juliet Adeola, Ifeyinwa Onwuatuelo, Ingrid V. Bassett, Elena Losina, Kenneth A. Freedberg, Prosper Okonkwo, Susan Regan

**Affiliations:** Division of Infectious Disease, Massachusetts General Hospital, 50 Staniford Street, 9th Floor, Boston, MA USA; Medical Practice Evaluation Center, Massachusetts General Hospital, Boston, MA USA; AIDS Prevention Initiative in Nigeria (APIN), Abuja, Nigeria; Division of General Internal Medicine, Massachusetts General Hospital, Boston, MA USA; Harvard University Center for AIDS Research (CFAR), Boston, MA USA; Department of Orthopedic Surgery, Brigham and Women’s Hospital, Boston, MA USA; Departments of Biostatistics and Epidemiology, Boston University School of Public Health, Boston, MA USA; Department of Health Policy and Management, Harvard T.H. Chan School of Public Health, Boston, MA USA

**Keywords:** Retention, Unplanned care interruption, Gaps in care, Resource-limited setting, HIV

## Abstract

**Background:**

Unplanned care interruption (UCI) challenges effective HIV treatment. We determined the frequency and risk factors for UCI in Nigeria.

**Methods:**

We conducted a retrospective-cohort study of adults initiating antiretroviral therapy (ART) between January 2009 and December 2011. At censor, patients were defined as in care, UCI, or inactive. Associations between baseline factors and UCI rates were quantified using Poisson regression.

**Results:**

Among 2,496 patients, 44 % remained in care, 35 % had ≥1 UCI, and 21 % became inactive. UCI rates were higher in the first year on ART (39/100PY), than the second (19/100PY), third (16/100PY), and fourth (14/100PY) years (*p* < 0.001). In multivariate analysis, baseline CD4 > 350/uL (IRR 3.21, *p* < 0.0001), being a student (IRR 1.95, *p* < 0.0001), and less education (IRR 1.58, *p* = 0.001) increased risk for UCI. Fifty-five percent of patients with UCI and viral load data had HIV viral load > 1,000 copies/ml upon return to care.

**Discussion:**

UCI were observed in over one-third of patients treated, and were most common in the first year on ART. High baseline CD4 count at ART initiation was the greatest predictor of subsequent UCI.

**Conclusions:**

Interventions focused on the first year on ART are needed to improve continuity of HIV care.

## Background

HIV is now a treatable, chronic disease with the availability of potent ART [1]. Accordingly, the strategic focus guiding the global HIV response has transitioned from acute crisis to chronic disease management [2]. Retention in care over time has plagued HIV treatment programs worldwide, particularly those in resource-limited settings (RLS) [3–5].

Retention in care requires a commitment to a package of clinical care including clinic appointments, laboratory visits, medication pick-up, and medication adherence. The early literature on retention in HIV care indicates startling rates of patient loss to follow-up (LTFU) in RLS, approaching 70 % prior to ART initiation and 25-40 % after ART initiation [5, 6]. Because many of these patients (15-90 %) are later found to have died, LTFU has been equated with disengagement from the entire package of HIV care [6–9].

It is now clear that patients defined as lost to follow-up represent many types of patients; some die, some voluntarily withdraw from care, others transfer care to different treatment facilities, while others interrupt but later return to care [10–15]. Patients with unplanned care interruptions (UCI) are at increased risk for CD4 decline, uncontrolled viremia, opportunistic infections, and long-term mortality [11, 13, 15–18].

The largest review of interruptions in care and treatment among HIV-infected patients highlights that such interruptions are common, occurring in approximately 25 % of patients initiated on ART [15]. This review also describes wide variation in definitions of care interruption ranging from 1 day to 1 year or more without treatment or care [15]. Risk factors for UCI in HIV care vary in different settings, however studies in RLS remain sparse [11, 15, 16, 19]. Nigeria is the most populous country in Africa, and has the second largest HIV-infected population, behind South Africa [20, 21]. Our objective was to determine the rates and risk factors for UCI among patients who have initiated ART in Nigeria.

## Methods

### Setting

This study was conducted at the Ahmadu Bello University Teaching Hospital’s (ABUTH) HIV clinic. ABUTH is located in a semi-urban community in the northern Nigerian state of Kaduna, HIV prevalence in the state is 9.2 % [20, 22]. The ABUTH clinic began providing care in 2006. In the same year, PEPFAR support commenced and ART was provided free of charge to all eligible patients. ABUTH currently offers comprehensive HIV testing and treatment to nearly 4,000 patients. During the study period, the clinic was managed by the AIDS Prevention Initiative in Nigeria (APIN), a PEPFAR-supported NGO, which is one of the largest HIV treatment programs in Nigeria.

### Study design

We conducted a retrospective cohort study of ART eligible patients ≥14 years of age enrolled in the ABUTH clinic, who initiated ART between January 1, 2009 and December 31, 2011. Patients were followed through December 31, 2012 to allow for a minimum of 1-year of observation for all patients. Women who were pregnant at enrollment or became pregnant during the follow-up period were not included in the analysis, as the protocol for clinic follow-up differed between this group and the general adult population. Upon clinic enrollment, patients received a variety of services according to protocol including clinical evaluation, TB symptom screening, adherence counseling, and CD4 count as well as viral load testing. Information on receipt of adherence counseling was not available in the electronic medical record. In the first 12 weeks after enrollment, patients were scheduled to be seen at 2, 4, 8, and 12 weeks for adherence counseling, clinical examination, and TB symptom screening. Subsequently, they were seen for ART pick-up every 4 weeks, clinical examination and adherence counseling every 12 weeks, and laboratory testing (including CD4 count and HIV viral load) every 24 weeks [23]. All data, including baseline demographic information, clinical visits and evaluations, laboratory visits and results, and drug pick-up visits were collected in structured data entry forms which were uploaded to APIN’s central electronic clinical database.

### Outcome measures

At study censor date (December 31, 2012) we categorized patients into 1 of 3 mutually exclusive groups based on their visit patterns. A visit was defined as any encounter with the clinic, whether for clinical, laboratory, or pharmacy services. Patients were defined as *in care* if the time between any two consecutive visits was ≤90 days, and the time between the last visit and censor date was ≤180 days. Patients were defined as having an *unplanned care interruption* (UCI) if the time between any two consecutive visits was ever >90 days, but they returned to clinic before the censor date. This definition was not dependent upon the time between the last visit and censor date. Finally, patients were defined as *inactive* if the time between any two consecutive visits was ≤90 days, but the time between the last visit and the censor date was >180 days. Patients known to have transferred care or died during the follow-up period were categorized based on their visit patterns prior to transfer or death (*i.e.,* such patients could not have been classified as inactive). Under routine circumstances, an absence from the clinic of at least 90 days implied that a patient missed three ART pick-up visits, and at least one clinical visit. In select circumstances, clinic protocol permitted dispensing of 2-month ART prescriptions to virologically suppressed patients on ART for >1 year. We chose a 90-day window to define UCI to ensure no overlap with this select group of stably suppressed patients.

### Statistical analysis

#### Rates of UCI and time on ART

We determined the ratio of the number of patients with at least one UCI in the first, second, third, and fourth years on ART; and the total person-time at risk for UCI during each year. We calculated rates of UCI at the end of each year on ART.

#### Predictors of UCI in the first year on ART

We built bivariate and multivariate Poisson regression models to assess the association between baseline age, sex, education, employment, TB diagnosis, CD4 count, and enrollment year on the rate of first UCI during the first year on ART. We focused the analysis on predictors of UCI in the first year on ART since other studies have linked early missed visits to mortality, and to ensure consistent follow-up time for all patients in the analysis [13, 16, 18]. Additionally, we recognized that patients with UCI early after ART initiation might be different from those who interrupt later; our goal was to investigate predictors of the former. Data were censored at the date of last visit (before first UCI or becoming inactive from clinic) or one year after ART initiation for those who remained in care. Covariates with associations at *p* ≤ 0.10 in the bivariate model were included in the final multivariate model. The multivariate model was adjusted for enrollment year to account for possible temporal trends that might impact UCI.

#### Change in CD4 count before and after UCI

We conducted a Wilcoxon matched-pairs signed rank test on available CD4 counts drawn at baseline, prior to UCI, and within 3 months of return from UCI for patients who interrupted care.

#### HIV viral load after UCI

We reported dichotomized viral loads within 3 months of return from UCI (>1000 copies/mL vs. ≤1000 copies/mL) for patients who interrupted care and had available data; baseline HIV viral load testing is not routinely performed in this population.

Statistical analysis was conducted with Stata Statistical Software (StataCorp. 2013. *Stata Statistical Software: Release 13*. College Station, TX, USA).

### Sensitivity analysis

There is substantial variation in how absences from HIV care are defined in the literature [15, 24]. Consequently, we varied the definition of UCI from 90 days to 180 days without clinic contact in sensitivity analysis.

### IRB approval

IRB approval was obtained from Partners HealthCare and Harvard T. H. Chan School of Public Health in Boston, MA, USA, and the Nigerian Institute for Medical Research in Lagos, Nigeria.

## Results

### Description of cohort

Our cohort was composed of 2,496 adults who enrolled at ABUTH and initiated ART between 2009 and 2011. Median observation time was 22.5 months [IQR 10, 36], and total person-years accrued during follow-up was 4,717. Females made up 69 % of the cohort and the median age was 32 years [IQR 27, 39]. Many patients (60 %) were married, and a large majority (77 %) had at least primary education. Sixty-three percent of patients were employed, 29 % were unemployed or retired, and 8 % were students. Sixty-two percent of patients presented with WHO stage 3 or 4 disease, and median baseline CD4 count was 207 cells/uL^3^ [IQR 98, 344] prior to ART initiation. Four percent of patients had TB upon enrollment (Table 1).

**Table 1:** Baseline demographics in a Nigerian cohort of HIV-Infected persons initiating ART between 2009 and 2011

	All		In care		Unplanned care interruption		Inactive	
	*n* = 2,496		*n* = 1,091		*n* = 867		*n* = 538	
	%	*N*	%	*N*	%	*N*	%	*N*
Sex								
Male	31	782	28	311	31	267	38	204
Female	69	1714	72	780	69	600	62	334
Median age (yrs) [IQR]	32	[27, 39]	32	[27, 40]	31.0	[26, 37]	32	[27, 40]
Marital status								
Married	60	1506	64	699	59	516	54	291
Single	26	641	21	230	28	243	31	168
Widowed	14	349	15	162	13	108	15	79
Employment								
Employed	63	1551	29	316	29	251	28	146
Unemployed/Retired	29	713	65	706	61	512	64	333
Student	8	201	5	63	10	94	8	44
Education								
Tertiary	32	804	34	373	30	260	32	171
Primary/Secondary	45	1119	43	468	48	414	44	237
None	23	573	23	250	22	193	24	130
Relative enrolled in ABUTH clinic								
No	82	2037	80	871	81	699	87	467
Yes	18	459	20	220	19	168	13	71
WHO stage baseline								
1	16	312	15	151	18	119	11	42
2	22	448	23	224	24	152	18	72
3	36	734	38	381	34	219	34	134
4	26	533	24	232	24	154	37	147
Baseline CD4/uL								
Median [IQR]	207	[98, 344]	193	[91, 291]	272	[144, 436]	172	[74, 289]
<50	13	269	14	125	10	63	18	81
50-100	12	247	13	119	9	61	15	67
101-200	23	457	25	227	17	116	25	114
200-350	28	566	33	297	24	162	23	107
>350	24	490	15	138	40	265	19	87
Baseline tuberculosis								
Yes	4	97	4	43	4	32	4	22
Enrollment year								
2009	39	970	35	379	46	400	35	191
2010	34	840	32	354	33	288	37	198
2011	27	686	33	358	21	179	28	149

*IQR* Inter-quartile range, *ABUTH* Ahmadu Bello University Teaching Hospital

Twenty-four percent (n = 490/2029) of patients in the cohort (with available baseline CD4 values) enrolled and initiated ART with CD4 > 350 cells/uL. We conducted a chart review to determine the reasons for ART initiation in this group. All were documented to have met national guideline criteria for starting ART. The indication for treatment was advanced WHO stage (3 or 4) in 36 %, continuation of ART for patients previously treated in 20 %, concomitant TB in 5 %, and co-infection with Hepatitis B virus in 3 % of patients. The indication for ART initiation was not documented in 36 % of patients.

### Patterns of care

**Fig. 1:**
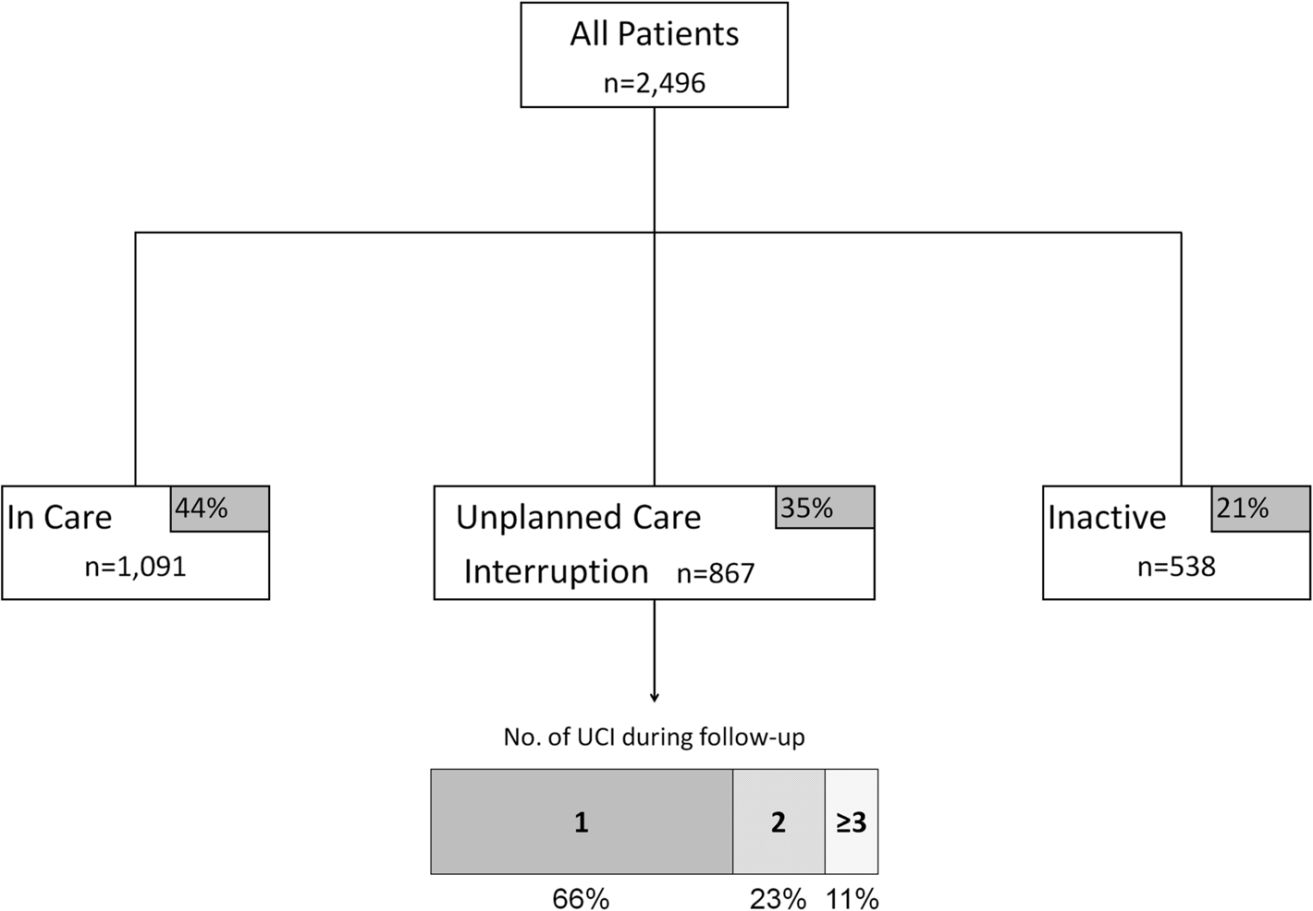
Summary of patient outcomes in a large HIV clinic study in Nigeria. *UCI* Unplanned care interruption

**Fig. 2:**
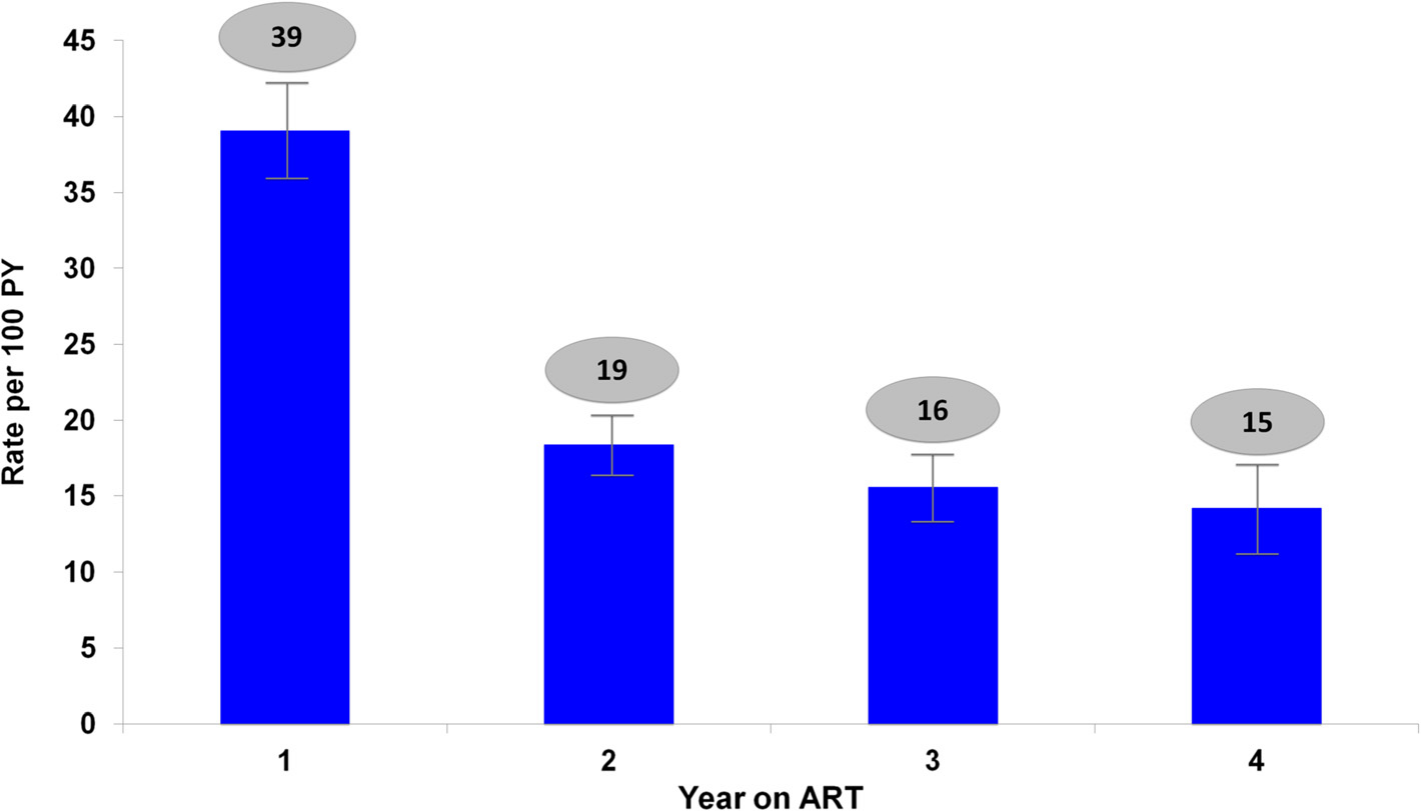
Rates of Unplanned Care Interruption by year on ART. *PY* person-year, *ART* antiretroviral therapy

At the end of follow-up, 44 % (*n* = 1,091) of patients remained continuously in care, 35 % (*n* = 867) had ≥1 UCI, and 21 % (*n* = 538) became inactive. Data about documented clinic transfers revealed that 4 % (*n* = 42) of patients who were in care, and 3 % (*n* = 31) of patients with UCI later transferred out of the clinic. Of the patients with UCI, 66 % (*n* = 576) had one interruption, 23 % (*n* = 198) had 2 interruptions, and 11 % had ≥3 interruptions (Fig. 1). UCIs lasted a median of 132 days [IQR 102, 205] before return to clinic. Patients remained in care a median of 114 days from enrollment before the first interruption in care [IQR 24, 364], and a median of 95 days [IQR 30, 238] before becoming inactive. We observed the highest rate of UCI in the first year on ART (39/100 PY, 95 % CI: 36–42). This decreased by more than half in the second year on ART (19/100 PY, 95 % CI: 17–21.0) and continued to decline in the third (16/100 PY, 95 % CI: 13–19) and fourth (15/100 PY, 95 % CI: 11–20) years on ART (Fig. 2).

### Factors associated with UCI in the first year on ART

In bivariate analysis, demographic characteristics associated with increased rate of UCI were being single (IRR 1.28, *p* = 0.007), having no education (IRR 1.21, *p* = 0.080), or primary/secondary education (IRR 1.22, *p* = 0.031) compared to tertiary education, and being a student (IRR 1.46, *p* = 0.007). Each decade increase in age was associated with a 15 % decreased risk of UCI (IRR 0.85, *p* < 0.0001). Patients who enrolled in clinic with CD4 count >350 cells/μL had an increased risk of UCI (IRR 3.30, *p* < 0.0001). Baseline CD4 count was lowest in the patients who later became inactive [median 172 cells/μL, IQR: 74, 289], and highest in those who later had interrupted care [median 272 cells/μL, IQR: 144, 436]. The risk of UCI did not significantly change with enrollment year (Table 2).

**Table 2:** Predictors of unplanned care interruption in the first year on ART in a Nigerian cohort^+^

	Bivariate analysis			Multivariate analysis		
	IRR	*p* value	95 % CI	IRR	*p* value	95 % CI
Sex						
Female	Ref					
Male	1.09	0.301	0.92, 1.29			
Age^a^	0.85	<0.0001	0.77, 0.93	1.06	0.333	0.94, 1.19
Marital status						
Married	Ref			Ref		
Single	1.28	0.007	1.07, 1.52	1.17	0.196	0.93, 1.45
Widowed	0.86	0.221	0.67, 1.10	0.82	0.174	0.61, 1.09
Employment						
Unemployed	Ref			Ref		
Employed	0.87	0.129	0.73, 1.04	1.10	0.417	0.88, 1.37
Student	1.46	0.007	1.11, 1.92	1.95	<0.0001	1.36, 2.80
Education						
Tertiary	Ref			Ref		
Primary/Secondary	1.22	0.031	1.02, 1.47	1.39	0.005	1.10, 1.75
None	1.21	0.080	0.98, 1.51	1.58	0.001	1.20, 2.09
Relative enrolled in same clinic						
No	Ref					
Yes	0.98	0.840	0.80, 1.19			
Baseline CD4/uL						
<50	Ref			Ref		
50-100	0.85	0.446	0.55, 1.30	0.77	0.243	0.50, 1.19
101-200	0.77	0.171	0.53, 1.12	0.72	0.085	0.50, 1.05
200-350	0.85	0.355	0.59, 1.21	0.81	0.241	0.57, 1.15
>350	3.30	<0.0001	2.40, 4.54	3.21	<0.0001	2.32, 4.44
TB baseline						
No	Ref					
Yes	1.11	0.589	0.75, 1.65			
Enrollment year						
2009	Ref			Ref		
2010	0.99	0.876	0.82, 1.18	0.88	0.251	0.71, 1.09
2011	0.94	0.510	0.77, 1.14	0.80	0.061	0.64, 1.01

*ART* Antiretroviral Therapy, *IRR* Incidence Rate Ratio

^a^Age is modeled in 10-year increments

^**+**^n = 2,000 with complete data on all variables in multivariate model

We conducted a multivariate analysis including variables significant in the bivariate analyses. A total of 467 patients (19 %) were excluded from the multivariate analysis due to unknown baseline CD4 count (158/632 with and 309/1864 without a care interruption). A further 33 patients (1 %) were excluded due to missing values for other variables in the model. In this analysis, having a high baseline CD4 count (>350 cells/μL) was associated with the greatest risk of UCI in the first year on ART (IRR 3.21, *p* < 0.001). Students (IRR 1.95, *p* < 0.0001) and patients with no education (IRR 1.58, *p* = 0.001) or primary/secondary education (IRR 1.39, *p* = 0.005) remained at increased risk for UCI in the multivariate model. While not statistically significant, there appeared to be a trend towards reduced risk of UCI among patients who enrolled in care in 2011 compared to those who enrolled in 2009 (IRR 0.08, *p* = 0.061) (Table 2).

The study results remained robust to different definitions of UCI (ranging from 90 to 180 days with no clinic contact) and inactive care (ranging from 90 days to 365 days between study censor date and last visit date).

### Immunologic and virologic response among patients with unplanned care interruption

Baseline CD4 counts were available for 77 % (*n* = 667) of all patients with UCI and follow up CD4 values were available for 82 % of patients after first UCI. Two-thirds of CD4 counts were within 3 months of return to care. Among patients with UCI with CD4 data available median CD4 count increased from 255 cells/μL [IQR 136, 429] at baseline to 358 cells/uL [IQR 185, 511, *n* = 590], (*p* < 0.0001) before the first UCI and decreased to 329 cells/μL within 3 months of return to clinic [IQR 194, 487, *n* = 459], (*p* = 0.0001). Viral load data was available for 72 % (*n* = 620) of patients with UCI, nearly half were within 3 months of return to care. Fifty-five percent of viral loads obtained within 3 months of return to care were >1000 copies/mL.

## Discussion

Missed visits from HIV clinic resulting in interruptions in HIV care, particularly in the first year on ART, are known to be a predictor of subsequent morbidity and mortality [13, 15, 16, 18]. In our study of almost 2,500 patients who started ART in Nigeria, nearly 1 in 4 had UCI, and another 1 in 5 became inactive from clinic. We found that patients who started ART with high baseline CD4 count had a greater than three-fold increased risk of UCI. Students, 72 % of whom were university matriculates, had a 2-fold increase in UCI. Additionally patients with less than tertiary education had a 50 % increased risk of UCI. More than half of patients with UCI for whom we had viral load data returned to clinic with virologic failure, as evidenced by viral load >1000 copies/mL.

There is a brief window of opportunity for clinics to identify and intervene on behalf of patients at increased risk for poor outcomes due to missed visits. We found that patients were generally in care for only 3 to 4 months before having an UCI. Furthermore, 12 % of patients with UCI in this cohort later became inactive from clinic. While a systematic review of data from Sub-Saharan Africa has shown that overall attrition rates are highest in the first year on ART, we believe this study is among the first to document that UCI is also greatest in the first year on ART (39/100 person-years) [6]. In our analysis, this rate declined by more than half after the first year on ART. Concomitant with high rates of interruptions in care, multiple studies from resource-limited settings also describe higher early mortality among patients on ART compared to patients in resource-rich environments [25–28]. These findings together underscore the importance of a rigorous focus on early, effective retention in care for patients initiating ART.

Our data did showed a possible trend towards improvement in the risk of UCI over time in the context of local and national efforts to improve retention. Nigeria’s 2010 National HIV guidelines called for “creation of effective linkage and retention mechanisms to maximize benefits of HIV treatment and care,” but did not provide specific recommendations for programs [23]. In 2011, the APIN network instituted a patient tracking protocol at all of its comprehensive sites, including ABUTH. APIN’s protocol calls for a multidisciplinary outreach team to identify patients after they miss a single clinic visit, contact them by telephone or home visit to determine the reason for the missed visit, and schedule a return visit. Additional follow-up is necessary to determine the impact of interventions such as these on retention and UCI.

Both feeling well and feeling unwell have been implicated as reasons for poor clinic attendance and LTFU in resource-limited settings [14, 15, 29]. Approximately 25 % patients in our cohort were initiated on ART with baseline CD4 > 350 cells/μL. Only 28 % of those were stably in care during the follow-up period; 54 % had interrupted care, and 18 % became inactive. These patients had a variety of indications for initiating ART, including advanced WHO stage, baseline TB, and concomitant hepatitis B virus infection. Nonetheless, adjusting for other demographic factors and time on ART, this group still had the greatest risk of UCI. In a systematic review of cohorts in Sub-Saharan Africa, between 31 % and 95 % of patients who have not yet met criteria to initiate ART are retained in care between enrollment in care and becoming ART eligible [5]. These patients would by definition have CD4 counts above current guidelines for ART initiation. They may feel well, and be less invested in care. Some programs have developed pre-ART care packages to improve retention in this group [23, 30]. Our findings, however, highlight poor retention even among patients with high CD4 counts who have already initiated ART. This has important implications for retention efforts as HIV treatment programs operationalize guidelines for ART initiation at higher CD4 thresholds, and some are calling for “test and treat” models regardless of CD4 count [31–34].

Several studies have shown increased risk of LTFU among single patients relative to their married counterparts [35–37]. In our analysis, single patients were at increased risk for UCI in bivariate, but not multivariate analysis. While it is possible that single patients may have less reliable support systems, and may be at greater risk for stigma and disruptions in care, ethnographic studies show that the relationship between social networks, stigma, and disclosure are complex [38]. Some studies highlight the importance of social networks and social capital to help mobilize resources for and commitment to care and treatment [39, 40]. On the other hand, a study from Malawi found that 74 % of patients who defaulted from care had not disclosed their HIV status to anyone in the household [29]. Other studies have underscored the challenges with trust, communication, discrimination and stigma faced by patients in both sero-concordant and sero-discordant relationships, often making it difficult to look to a marital or sexual partner for consistent adherence support [41–43]. These studies and others suggest that the promise of social support is balanced by fear of being ostracized morally, economically, and socially [38, 44].

We also identified a novel patient group, students, at greater risk for inconsistent care. Nearly 3 in 4 students in our cohort had UCI or became inactive from the clinic. At least 1 in 10 Nigerian youth are enrolled in secondary or tertiary educational programs [45]. This age group (15–29 year-olds) has the highest prevalence of HIV infection, and accounts for nearly one-third of Nigeria’s population [46, 47]. While there is a growing body of literature suggesting poorer treatment outcomes and retention among HIV-infected adolescents compared to their adult counterparts, the student population has received little attention [48, 49]. In our analysis, age was not a significant predictor of UCI in the first year on ART, even when categorized to compare adolescents to young and older adults. Many reasons may contribute to inconsistent care among students, including challenges in transitioning from home to school, adjustment to new HIV care providers (for those infected prior to starting university schooling), absence of a trusted support system, and frequent disruptions in the academic schedule by faculty and staff strikes in Nigeria [50, 51]. During these strikes, students may leave the campus for weeks or months to return home or to other family or friends [51]. Understanding barriers to care for students is an important next step to ensure successful clinical outcomes for this group.

The obstacles to consistent care may be different between resource-limited and resource-rich environments. In our study patients with less than tertiary-level education had a substantial increase in their risk of UCI. In a country with 23 % unemployment, it is likely that tertiary education is a proxy not only for formal education, but also or greater individual or household resources [52]. In Nigeria and other resource-limited settings, ART pick-up is tied directly to the clinic. While the medications themselves may be free, the cost of getting to the clinic or pharmacy and competing priorities have been identified as sometimes insurmountable challenges to effective adherence to care [53–55]. In our cohort, 93 % of all clinical encounters involved ART pick-up. In contrast, in the United States and other well-resourced environments, patients often have the option to retrieve ART prescriptions at pharmacies with convenient hours located close to home, or delivered directly to the home. Ninety percent of Americans live within five miles of a community pharmacy [56]. One qualitative study investigating reasons for interruptions in care in Nigeria, Tanzania, and Uganda described a level of unpredictable chaos that routinely disturbs patients’ plans and schedules [12]. The authors characterized this chaos as one of the unintended reasons for UCI. One consideration to reduce this obstacle is more flexible ART dispensation (>30 days) to reduce the burden of clinic visits earlier in the course of ART. In APIN programs, longer ART prescriptions may be made available, but typically only to patients who are stable on ART with continued virologic suppression after one year.

This study has several limitations. The cohort reflects one hospital-based clinic site in Nigeria, which may not be fully generalizable to other regions in Nigeria or other resource-limited settings. Because of the retrospective design, we were not able to assess reasons for UCI, including whether some temporarily transferred care, or to assess outcomes among patients who became inactive from clinic. Follow-up CD4 count and viral load values were available for only a subset of patients, and thus our estimates of immunologic and virologic consequences of interrupted care may be biased. We did not have information about which patients received ongoing adherence counseling, nor did we have full data about patient deaths, and thus could not assess the relationship between unplanned care interruption and these factors.

This analysis also has several strengths. It is one of the first African studies outside of South Africa to identify predictors of unplanned interruptions from HIV care. The study was set in Nigeria, where 10 % of the global AIDS population resides [20]. Our analysis relied on the robust electronic database of APIN, which captures patient encounters within the clinic, laboratory, and pharmacy. We were therefore able to assess a range of clinic encounters to investigate rates and predictors of overall unplanned interruptions from HIV care. In addition, because we had documentation of ART pick-up from pharmacy records, we knew that missed pharmacy visits implied that patients were not taking ART dispensed from the ABUTH clinic.

## Conclusions

In conclusion, patients with consistent, stable engagement in HIV care are in the minority in a large ART program in Nigeria. Within the first four months after initiating ART, more than half of patients interrupt care for 90 days or more or become inactive from clinic. Given the impact of missed visits on subsequent immunologic and virologic compromise, as well as mortality, it is important to understand the reasons for interruptions in care. In our cohort, less educated patients, students, and those with higher baseline CD4 count were at greatest risk. Interventions to improve retention in care focused on high risk patients early after ART initiation are urgently needed.

## Abbreviations

ABUTH: Ahmadu Bello University Teaching Hospital
APIN: AIDS Prevention Initiative in Nigeria
ART: Antiretroviral therapy
LTFU: Loss to follow-up
RLS: Resource-limited settings
UCI: Unplanned care interruption
